# #ChronicPain: Automated Building of a Chronic Pain Cohort from Twitter Using Machine Learning

**DOI:** 10.34133/hds.0078

**Published:** 2023-07-04

**Authors:** Abeed Sarker, Sahithi Lakamana, Yuting Guo, Yao Ge, Abimbola Leslie, Omolola Okunromade, Elena Gonzalez-Polledo, Jeanmarie Perrone, Anne Marie McKenzie-Brown

**Affiliations:** ^1^ Department of Biomedical Informatics, School of Medicine, Emory University, Atlanta, GA, USA.; ^2^ Department of Radiology, Robert Larner College of Medicine, University of Vermont, Burlington, VT, USA.; ^3^ Department of Health Policy and Community Health, Jiann-Ping Hsu College of Public Health, Georgia Southern University, Statesboro, GA, USA.; ^4^ Department of Anthropology, Goldsmiths University of London, London, UK.; ^5^ Department of Emergency Medicine, Perelman School of Medicine, University of Pennsylvania, Philadelphia, PA, USA.; ^6^ Department of Anesthesiology, School of Medicine, Emory University, Atlanta, GA, USA.

## Abstract

**Background:**

Due to the high burden of chronic pain, and the detrimental public health consequences of its treatment with opioids, there is a high-priority need to identify effective alternative therapies. Social media is a potentially valuable resource for knowledge about self-reported therapies by chronic pain sufferers.

**Methods:**

We attempted to (a) verify the presence of large-scale chronic pain-related chatter on Twitter, (b) develop natural language processing and machine learning methods for automatically detecting self-disclosures, (c) collect longitudinal data posted by them, and (d) semiautomatically analyze the types of chronic pain-related information reported by them. We collected data using chronic pain-related hashtags and keywords and manually annotated 4,998 posts to indicate if they were self-reports of chronic pain experiences. We trained and evaluated several state-of-the-art supervised text classification models and deployed the best-performing classifier. We collected all publicly available posts from detected cohort members and conducted manual and natural language processing-driven descriptive analyses.

**Results:**

Interannotator agreement for the binary annotation was 0.82 (Cohen’s kappa). The RoBERTa model performed best (F_1_ score: 0.84; 95% confidence interval: 0.80 to 0.89), and we used this model to classify all collected unlabeled posts. We discovered 22,795 self-reported chronic pain sufferers and collected over 3 million of their past posts. Further analyses revealed information about, but not limited to, alternative treatments, patient sentiments about treatments, side effects, and self-management strategies.

**Conclusion:**

Our social media based approach will result in an automatically growing large cohort over time, and the data can be leveraged to identify effective opioid-alternative therapies for diverse chronic pain types.

## Introduction

Between 5.5% and 33% of the world’s and between 11% and 30% of the United States (U.S.) adult population are estimated to suffer from chronic pain [1–4]. The total financial burden of chronic pain in the U.S. is estimated to be between $560 and $635 billion per year [5]. Opioids have remained the go-to treatment for years [3], but they are addictive and they have been credited with heavily contributing to the epidemic of drug overdose-related deaths in the U.S., particularly in the early years. Due to the enormous financial and nonfinancial cost of the use of opioids as first-line therapy for chronic pain, recent research efforts have focused on identifying alternative treatments, including pharmacological, natural, physical, and behavioral therapies [6]. Currently, evidence is limited regarding the efficacy of many alternative therapies [6–10], the associations between types of pain and effective alternative management strategies, and the risks involved with the different strategies (e.g., long- and short-term side effects). It is difficult to establish the evidence and risk profiles of alternative therapies as they require studies in clinical settings, particularly trials. However, many alternative therapies have widespread use, and curating the knowledge from peoples’ experiences in a systematic manner may lead to the discovery of useful alternative therapies for targeted chronic pain types.

Many recent studies have leveraged social media big data for obtaining insights directly from patients about targeted health-related topics including, but not limited to, substance use [11–14], adverse drug reactions [15–17], and mental health [18–20]. Over 220 million Americans (~70%) use social media, and there is an abundance of health-related information on such platforms [21,22]. Recent advances in data science and machine learning have enabled researchers to extract complex information expressed in natural language. These advances have made it possible to curate specialized cohorts from social media for conducting longitudinal studies based on data posted by the cohort members, such as breast cancer patients [23], pregnant people [24], and people who use substances nonmedically [25]. Social media thus present an attractive and underexplored opportunity for studying people suffering from chronic pain at low cost, unobtrusively, and at scale, provided that a chronic pain cohort can be built automatically from such sources, and longitudinal data from the cohort members can be collected and analyzed.

Several past studies have demonstrated the utility of social media data for chronic pain-related research. Some early studies focused on studying the impacts of social media on chronic disease management, rather than the use of it to study chronic pain specifically [26,27]. Through a global online survey conducted partially via social media, Merolli et al. [27] concluded that areas of research that warranted attention included the ability to (a) filter information and guide people to pertinent information, (b) connect sufferers of chronic pain (i.e., cohort members), and (c) explore relationships between the therapeutic affordances of social media and health outcomes. In a separate, questionnaire-based study, Ressler et al. [28] found that posting about chronic pain online may decrease the sense of isolation and increase a sense of purpose. Similar findings were reported by Tsai et al. [29] in a more recent study. Gonzalez-Polledo et al. [30] studied social media (Tumblr and Flickr) and chronic pain from the perspective of digital anthropology, characterized chronic pain narratives over these platforms, and presented a typology of chronic pain expressions.

Works most closely related to ours are those by Sendra and Farre, and Mullins et al. [31,32]. In the former study, the authors analyzed a small sample of data (*n* = 350) and concluded that social media is changing the way patients live with their chronic pain and care providers could benefit from paying attention to self-reported information by these individuals. Mullins et al. collected a small sample of data from Twitter and performed natural language processing (NLP)-driven analysis of discussion topics, sentiments, and advice provided. The study concluded that the pain-related discussions on the platform can enrich our understanding of the chronic pain experience.

In this paper, we take the first steps toward building a system that automatically detects self-reports of chronic pain from social media, specifically Twitter. Our study (a) verifies that reports of chronic pain are frequently posted on Twitter by sufferers themselves, (b) such reports can be automatically detected via NLP and supervised machine learning, and (c) longitudinal data posted by self-reported sufferers of chronic pain contain a trove of knowledge about chronic pain therapies, including nontraditional therapies and self-management strategies, their efficacies, and risks. We focus this paper on the chronic pain cohort-building process, leaving the tasks of mining longitudinal data and deriving potential causal associations as future work. The automatically detected cohort will continue to grow over time and will enable us to study a large group of people over many years. Our annotated dataset is made publicly available with this publication (see supplementary material) in order to enable interested researchers to reproduce our work and deploy the pipeline for their own analyses.

The rest of the paper is organized as follows: in Methods, we describe the data collection, manual annotation, supervised classification, and postclassification analyses (sentiment and content analyses) approaches; the outputs from these approaches are presented in Results; in Discussion, we outline the utility of our approaches for creating and data from a chronic pain cohort from social media, discuss errors made by the supervised classification system and limitations of the current work, propose future work, and present some concluding remarks.

## Methods

**Fig. 1.**
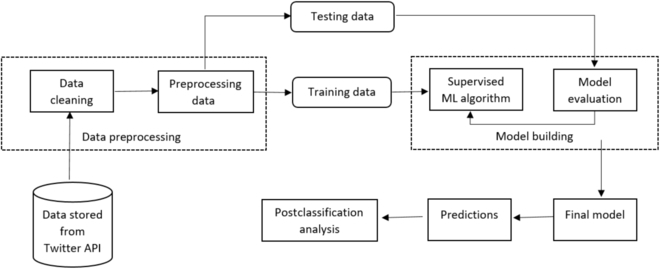
End-to-end data processing framework involving data collection/storage via the Twitter API, data preparation and preprocessing, training, evaluation, and classification using the final model and postclassification analyses.

This study was reviewed by the Institutional Review Board of Emory University and considered to be exempt (category 4; publicly available data). Figure 1 presents our data processing pipeline for this study. The overall study can be divided into 4 steps: (a) data collection, (b) manual annotation, (c) supervised classification, and (d) postclassification analyses. We now describe each of these steps.

### Data collection

We collected data through the Twitter academic streaming application programming interface (API) [33]. The API enables the collection of a sample of data in real time based on keywords. We used the hashtag “#chronicpain” and the phrase “chronic pain*”*. For the phrases, we first collected tweets using the terms individually and then applied a regular expression pattern to detect the exact phrase. We excluded retweets and tweets that did not meet this exact expression.

### Annotation

We randomly selected 5,000 posts from our entire collected set for manual annotation. The objective of the annotation was to determine whether a post represented a personal experience or not. Thus, the annotation was a binary labeling task indicating if a tweet represented a self-report (label: Y) or something other than a self-report (label: N). Label N represented tweets that were general information about chronic pain or those that could be about raising awareness or promotion. We conducted the annotation in 3 batches. In the first batch, we annotated a small sample of tweets (*n* = 200) without an explicit guideline and we discussed the disagreements from this round of annotation to create a simple annotation guideline. We annotated a larger sample (*n* = 1,300; batch 2) following this guideline, and we further discussed disagreements and resolved them. We made minor updates to the annotation guideline based on these discussions. Since the disagreements were relatively low after the second round of annotations, we annotated the remaining tweets (batch 3).

Randomly selected subsets of the tweets were annotated by 3 annotators. We used these overlapping annotations to compute interannotator agreements (IAA). Since the interpretation of the tweets can be subjective, it was important to capture the extent of human agreement as it represents a potential ceiling for any machine learning algorithm used to automate the process. We used the Cohen’s kappa measure to measure IAA. All disagreements were resolved by the first author of the article (A.S.). The annotation guideline is available as supplementary material. Table 1 presents examples of tweets and their labels.

**Table 1.** Sample posts representing self-reported chronic pain (Y) and generic chronic pain-mentioning posts (N).

Post	Label
"A2. Being Black affects every aspect of my #ChronicPain experience. People don't believe my chronic pain experience. They perceive my pain as an over exaggeration. From doctors minimizing my chronic pain to assuming I can just ""endure"" it.	Y
Chronic pain will have you thinking the craziest thing. My hips are killing me so I thought it might be nice to not have legs…. But then I realise my back and arms also hurts so then I thought what about being a floating head… then I remembered migraines. #chronicpain	Y
26 years old, 15 years of chronic pain and fatigue... finally diagnosed with Endometriosis #awareness #endometriosis #ChronicPain	Y
Good luck with that!! I have 3 herniated discs, sitting on nerves, spinal hypertrophy, chronic pain...diagnosed 7 months ago and my appt to see an NHS specialist THIS Oct has been cancelled Now on the waiting list No end in sight!!! Hope u have better luck #ChronicPain	Y
My chronic pain is gotten worse since my covid infection. I wake up with pain in my neck muscles every day now.	Y
"Here is a good paper from The Lancet looking at chronic pain: an update on burden, best practices, and new advances. Doesn't look like it's open access but you might be able to access it from your institution. [link]	N
20 Tips: Making the Best of 20 Years of Chronic Pain and Illness [link] #chronicpain	N
"Plant-derived compound may help treat chronic pain. #chronicpain #CRP [LINK]"	N
"Chronic pain and low oxalate diet? Some people find a huge amount of relief from trying a low oxalate diet when they have chronic pain. Have you tried it? Let me know #chronicpain #spoonie #chronicillness #cfs #fibro"	N

### Supervised classification

We divided the annotated data into 3 sets—60% for training, 20% for validation, and 20% for testing/evaluation. Transformer-based approaches that use large pretrained language models such as BERT are currently state-of-the-art for text classification, both in and outside of the medical domain. We, therefore, fine-tuned and evaluated several transformer-based classifiers. The following is an outline of the classifiers we used:

1. RoBERTa: A transformer-based model popular for its training on big batches and long sequences [34].
2. SciBERT: This model is trained on a large corpus of scientific data and with the same model structure as BERT [35].
3. BioClinicalBERT: This is extended from BioBERT (BioBERT-Base v1.0 + PubMed 200K + PMC 270K) and is trained on MIMIC III notes [36].
4. BERTweet: A large-scale pretrained model for English tweets [37].
5. BioBERT: This is specifically trained for biomedical text and is widely used for biomedical text mining [38].
6. Clinical_KB_BERT: This is trained over BioBERT using a joint training method that adds knowledge base information into the model from the Unified Medical Language System [39].

We compared the performances of the classifiers based on the F_1_ score for the Y class. The F_1_ score is the harmonic mean of precision and recall. We focused our evaluation on the Y class since that is our class of interest. We also report overall classifier accuracy, but this metric is primarily driven by the majority (N) class. We computed the 95% confidence intervals for the Y class F_1_ scores using the bootstrap resampling technique [40]. In this method, the F_1_ score is calculated 1,000 times for randomly selected (with replacement) test set predictions. Out of these, 25th and 975th values are considered as lower bound and upper bound values at 95% confidence interval.

### Postclassification analyses

We used the best-performing classifier (in terms of F_1_ score) from the previous subsection to classify unlabeled posts collected from January 2021 to March 2022. Then, for a sample of subscribers whose posts were classified to be self-reports of chronic pain (Y), we collected all their past posts available via the API. The Twitter API allows the collection of approximately 3,200 past public posts by each subscriber. Thus, for many subscribers—members of our chronic pain cohort—this enabled us to obtain multiple years of posts. We were only able to collect this for a subset of the subscribers due to the API limitation of 10 million posts per month. Finally, we semiautomatically analyzed samples of the cohort posts to assess the presence and type of chronic pain-related information. To detect therapy-related information, we first created a lexicon of therapies, including medication and other therapies. Then, we applied a fuzzy matching approach [41] to detect potential therapies mentioned, including pharmaceuticals such as opioid medications (e.g., oxycodone and hydrocodone) and nonstandard ones such as behavioral therapies. Following these automatic steps, we performed a number of manual analyses involving the posts that mentioned specific therapies and also the past posts collected from the subscribers. We outline these below.

#### Therapy-related posts analysis

We extracted posts that were detected to mention at least 1 therapy and compared their distributions. We also drew a small sample of posts mentioning therapies and conducted a sentiment analysis to obtain an estimate of how subscriber sentiments were associated with each therapy, if at all, and if the data held potentially differentiable therapy-specific sentiments. Two coauthors (A.L. and O.O.) manually reviewed each post and determined whether the sentiment expressed in relation to the therapy was (a) positive, (b) neutral, or (c) negative. We chose a manual approach for the sentiment analysis, rather than an automatic one, because there is no resource (e.g., lexicon) that specifically caters to this task. Many keywords associated with chronic pain posts have negative sentiments (e.g., the term “pain” has a negative sentiment score in the VADER [42] lexicon), leading to sentiment estimates being biased. We compared the overall distributions of the mentioned therapies in our cohort data and also the distributions of sentiment associated with each therapy.

#### Cohort post content analysis

For Twitter subscribers who mentioned more than 3 chronic pain- or treatment-related posts, we also reviewed samples of their unlabeled tweets to identify other information relevant to chronic pain research and the topics of discussion. In-depth analyses of the types of information posted by the cohort and their quantification were considered to be outside the scope of this study. Instead, we simply focused on characterizing the types of relevant information present and verifying their contents for future analyses.

## Results

### Data and annotation

Of 5,000 posts selected for annotation, 2 were excluded due to issues with text encoding, leading to the annotation of 4,998 posts in total. A total of 550 posts were at least double annotated. Pair-wise IAA among 3 annotators was *k* = 0.82 (Cohen’s kappa [43]), which can be considered to be almost-perfect agreement [44]*.* A total of 719 (14.4%) were self-reports of chronic pain while the rest (85.6%) were not. The class distribution was thus slightly imbalanced. This was unsurprising since our past studies have found similar imbalances [11,45]**.**

### Classification performance

**Fig. 2.**
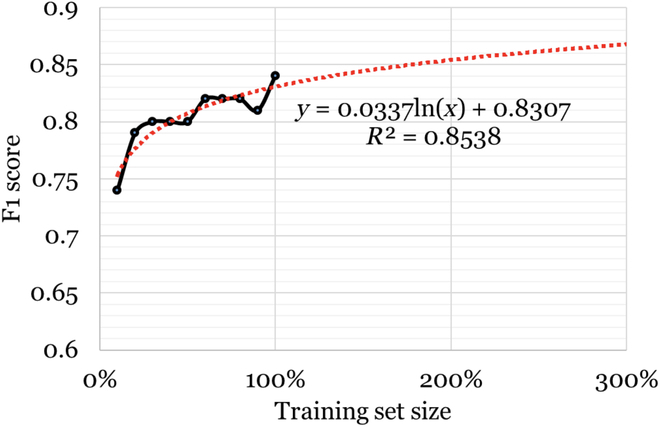
Classifier F_1_ scores or the positive (Y) class at different training set sizes and projected scores based on a logarithmic trendline for the RoBERTa model.

Table 2 presents the results of our automatic classification experiments. The table shows the overall accuracy of the classifiers, the F_1_ scores for the positive class and the 95% confidence intervals. The RoBERTa classifier achieved the highest F_1_ score among all the classifiers. For the best-performing classifier, we conducted ablation experiments using 10% subsets of the original training data. Figure 2 shows, and as is typically seen from such experiments, that the performance of the classifier increases approximately logarithmically as more training data is added. The best-fit logarithmic trendline suggests that F_1_ scores of 0.87 would require us to increase the annotated dataset by 3 times. Accuracy scores are relatively unchanged with training data size since this value is primarily driven by the majority negative class.

**Table 2.** Classifiers, their overall accuracies, F_1_ scores, and 95% confidence intervals (CIs) for the F_1_ scores.

Classifier	Accuracy	F_1_ score (Y class)	95% CI of F_1_ score
RoBERTa	0.95	0.84	0.80–0.89
SciBERT	0.93	0.79	0.74–0.84
BioClinicalBERT	0.95	0.82	0.76–0.86
BERTweet	0.95	0.83	0.78–0.87
BioBERT	0.95	0.82	0.76–0.86
Clinical_KB_BERT	0.94	0.80	0.74–0.84

### Postclassification analyses

The application of our classifier on the unlabeled posts resulted in the identification of 41,262 self-reports of chronic pain from 22,795 subscribers. Collecting their past data resulted in 3,461,619 posts (~151 posts per user on average). We performed NLP-driven and manual postclassification analyses on these posts, and we describe the findings in the following subsections.

#### Therapy-related post analysis

Table 3 presents the therapies we discovered from the cohort-posted data and their distributions. The therapies we discovered include prescription medications such as opioids, nonprescription pharmacological substances such as cannabidiol (CBD) and cannabis, physical therapies such as massage and chiropractic, and behavioral therapies such as meditation and yoga. We even discovered many examples of relatively unexplored therapies such as music therapy, aromatherapy, and guided imagery. Cannabis was the most commonly mentioned substance in the cohort timelines, although our manual review suggested that many of the posts were about recreational use rather than their use for treating chronic pain. A substantial number of tweets, however, did describe the use of cannabis-related products for treating chronic pain. Chiropractic was the most commonly mentioned physical/manual therapy. Table 4 presents examples of tweets mentioning therapies and their categories. As illustrated in the table, posts often describe self-management strategies for chronic pain, pharmacological substances, and their efficacies, comparisons between different pain management strategies, and adverse effects of therapies (e.g., opioid pain relievers) including long-term impacts (e.g., addiction).

**Table 3.** Therapies mentioned by our chronic pain cohort and their distributions in the longitudinal cohort data.

Treatment	Number of posts	Number of subscribers	Proportion of subscribers (%)
delta-8/weed/marijuana/cannabis/THC	21,829	12,973	56.9
CBD	6,312	3,923	17.2
Chiropractic	4,765	2,003	8.8
Yoga	3,162	1,773	7.8
Massage	3,148	1,701	7.5
Meditation	2,244	1,411	6.2
Acupuncture	1,919	540	2.4
Dietary	800	437	1.9
Relaxation	756	411	1.8
Hypnosis	483	288	1.3
Morphine	338	223	1.0
Buprenorphine	332	199	0.9
Methadone	214	138	0.6
Tramadol	178	98	0.4
Aquatic	166	103	0.5
Tai Chi	146	71	0.3
Aromatherapy	128	78	0.3
Music therapy	120	66	0.3
Oxycodone	108	51	0.2
Biofeedback	71	43	0.2
Hydrocodone	56	27	0.1
Guided imagery	34	11	0,1

**Table 4.** Sample posts relevant to chronic pain detected within the public timelines of our automatically built cohort. The posts present a plethora of information including the types and descriptions of chronic pain, therapies, social support, questions about therapy and chronic pain, self-management strategies, mental health, and treatment access (including health insurance coverage).

Post	Therap[y|ies]	Therapy type(s)
stopped my yoga for a couple of weeks and now my back is killing me again	Yoga	Behavioral/physical
I have been reducing my opioid dosage after over a decade of taking them for chronic pain (jaw/face) and struggling with them greatly at times. TODAY I AM OFFICIALLY OFF MORPHINE! No more Fentanyl, OxyContin etc. either! Today I am proud of myself!	Opioid	Pharmacological (prescription)
	Morphine	
	Fentanyl	
	Oxycontin	
First time I was prescribed Oxycontin, my doctor had me watch a video about how if you truly had chronic pain, you could not become addicted to Oxycontin.I became addicted to Oxycontin.Then I was told I didn't have *real* pain, because if I did, I couldn't have been addicted.	Oxycontin	Pharmacological (prescription)
Exercise and diet helped my neck pain way more than those pain meds	Pain meds	Pharmacological and behavioral/physical
	Exercise	
	Diet	
If it weren’t for canabis, I would still be addicted to opioids and my pain would never get better	Cannabis	Pharmacological
	Opioids	
'm a chronic pain patient. I used to take 3 big gun oxycontin a day and overuse my scripts. Today, I use cannabis which isn't chemically addictive and has impressive medical benefits for those genuinely sick and standing in need	Oxycontin	Pharmacological
	Cannabis	
Have you tried acupunctur? My chiro couldn’t help so I tried it & it worked wonders!! J	Acupuncture	Behavioral/physical
	Chiropractic	
Now I am trying CBD…. Will let ya know if it helps in any way	Cannabidiol	Pharmacological

#### Sentiment analysis

**Fig. 3.**
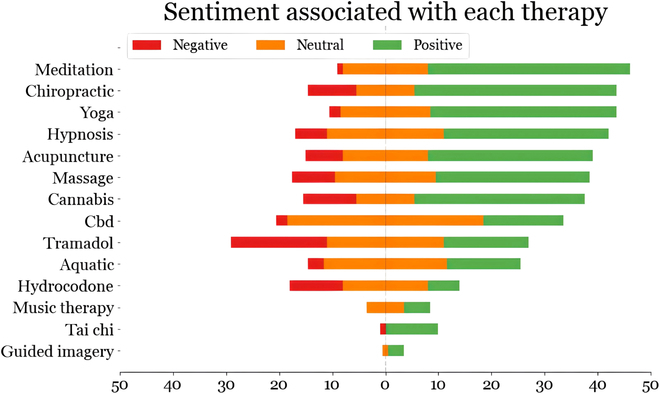
Distribution of manually annotated sentiments (positive, negative, and neutral) for different chronic pain therapies, as discussed by the cohort members.

Sentiment analysis is performed to estimate the polarity distribution of feelings associated with the chronic pain experienced by the cohort members who also mention specific treatment keywords. Six hundred posts were manually annotated in total with an IAA of 0.88 (Cohen’s kappa). Figure 3 presents the sentiment distributions for different types of therapies. The figure shows that most of the users who mentioned “meditation”, “Guided imagery”, and “Tai Chi” in their posts, also showed more positive emotions, while users who mentioned “hydrocodone” and “tramadol” show negative emotions compared to others.

#### Content analysis of cohort posts

Table 5 presents examples of posts from the longitudinal cohort data that present chronic pain-related information. The types of relevant information posted by the cohort include but are not limited to: descriptions of pain, therapies, impacts of therapies, self-management strategies, social support and its impact, mental health, treatment access-related information, and questions about chronic pain. The presence of such wide-ranging information suggests that publicly available data from this cohort may be invaluable in conducting long-term chronic pain-related studies. We further discuss the utilities of this data source and our data curation strategy in the following section.

**Table 5.** Sample posts relevant to chronic pain detected within the public timelines of our automatically built cohort. The posts present a plethora of information including the types and descriptions of chronic pain, therapies, social support, questions about therapy and chronic pain, self-management strategies, mental health, and treatment access (including health insurance coverage).

Post	Type of Information
Chronic pain does suck. Now that years of hip pain is gone I'm back to the lower back pain. Granted not near as bad as it was. I can control it thru movement/targeted exercise. But still have a day here n there that just suck. Getting old is not for the weak of spirit/humor.	Type and description of chronic pain
	Therapy
pain in my shoulders + back with an undiagnosed cause and i hate how often people tell me to go to a chiro!!!if several people with medical degrees can't figure me out, i definitely don't trust someone without one	Type and description of chronic pain
	Therapy
Years of oxy have made it worse and now on top of my pain I have lost everything	Type and description of pain
	Therapy
	Impact of long-term therapy (opioid)
I tried that too!! Tapered over months and no opioids wd… finding the right partner is key… thank you for your help	Self-management strategy
	Therapy
	Social support
my #chronicpain family has kept me going. Wud’ve given up way back otherwise	Social support
did a cold compresss help with the knee? Mine flares up after evry game	Question about therapy
Periodic reminder that corsetry is actually amazing, fixes back pain, keeps you from slouching, supports your boobs, and in my case, was a way to address mild body dysmorphia without risking my own tendency to obsess over things getting pointed at diet and exercise	Therapy
it took a toll on my #mentalhealth too.. never thought I would make it but now im clean for 3 months and rying alt treatments	Therapy
	Mental health
	Self-management
My insurance wont cover it though… so back to oain meds again and then they’ll treat me like a junkie. Y isn’t anyone doing anything about this??? #chronicpain	Therapy
	Insurance/treatment access

## Discussion

Our study verifies that social networks such as Twitter contain valuable information about chronic pain, posted by sufferers themselves. However, separating such information from the massive volumes of constantly generated data on Twitter is a challenging task, not manually achievable. Our methods automated the process of (a) detecting chronic pain-related posts automatically in real time, and (b) identifying posts from patients describing their own experiences. This strategy of automatic data collection and cohort curation will be able to establish a massive cohort over time and will enable us to study multiple years of data, including longitudinal data from the entire cohort and its subsets. This innovative strategy has additional advantages: (a) it allows us to include people who may not be reachable via traditional settings, such as hospital-based settings (e.g., because of lack of health insurance); (b) it is very cost-effective and is unobtrusive; and (c) it is able to continuously grow the cohort, thus enabling us to gather big data for long-term research. We now outline some related work in order to put our contributions into context. We then verify the utility of automatic classification approaches for monitoring and studying chronic pain. Finally, we present a brief discussion of errors to identify essential future improvements and we discuss some of the limitations of this study.

### Utility of social media

Our findings illustrate that social media is a rich source of information for studying chronic pain. Chronic pain-related discussions are common on Twitter, but this resource has thus far been underutilized in both epidemiological and interventional research. A prime reason for this is the difficulty of handling such massive data and processing complex language. Our proposed machine learning and NLP methods have the potential of overcoming the barriers leading to the underutilization of such an effective source of information. Successful deployment of this pipeline is likely to increase the utility of social media in chronic pain research. Our study also found that the Twitter chronic pain cohort discusses a variety of pain-related topics including but not limited to therapies, addiction, mental health, and support. Conducting more detailed analyses of texts associated with these topics may lead to important breakthroughs in chronic pain research. For example, analyses of the opioid alternatives discussed by subscribers can generate hypotheses about their efficacies, which can be tested in the future through more traditional research mechanisms.

Studying a social media-based cohort may also reveal information about factors impacting the sufferers of chronic pain that may not be available from any other source. These may include, for example, their social support (e.g., number of followers, number of interactions with a given post) and its influence (if any) on the quality of life of chronic pain patients. Data gathered by our methods may also enable researchers to connect alternative therapies and treatment strategies to social aspects of living with pain, ensuring their better efficacy. Monitoring the large cohort over time may lead to the discovery of social/therapeutic and efficacy-related aspects of alternative therapies that are not visible in traditional settings. The scale of knowledge that can be mined from this cohort is unparalleled, and the longitudinal nature of the data will enable the analyses of patient experiences and satisfaction in the short and long run. There is also the potential of going beyond epidemiological studies over social media and conducting interventional studies (e.g., reaching out to cohort members to suggest therapies, recruit for clinical studies, or to warn about potential adverse impacts of self-management strategies).

### Error analysis

We conducted a manual analysis of the misclassified tweets to identify patterns of errors. We focused on both false positives (classified erroneously as self-reports) and false negatives (classified as not self-reports). Under the former category, we found that most posts were misclassified when users intended to share the experiences of the people around them, not their personal experiences. One such post is, “today is mother's day and it's not an easy day. although my mom is still biologically alive, it feels like i've lost her to her chronic pain years ago. they say to treasure every moment but it's not always easy”. Under the latter category where model fails to classify as self-reports, we found that one common reason for misclassification was due to the expressions being implicit, making it difficult for the machine learning algorithms to capture their true meanings. One such example is, **“**music therapy for <hashtag> chronicpain. thinking of ways to make movement enjoyable might involve <hashtag> music <hashtag> dance. sometimes I'll just chuck on some of my favorites and dance, mindfully. i always feel a million bucks after as well as more connected with my body.” Another reason for misclassification in this category was determined to be incomplete information/partial information. For instance, “<hashtag> finally starting to ease, been trying to focus my mind on other things to get my brain to turn down the chronic <hashtag> pain it's at <number> out of <number>”*.* While for a human reviewer the post has enough context, it could not be picked up by the machine learning classifier.

### Limitations and future work

The most important limitation of the current method is perhaps the performance of the machine learning classifier. The F_1_ score for the positive class is not perfect (0.84), and there is room for improvement. Since most of the posts are not self-reports of chronic pain, the dataset is highly imbalanced—the negative class comprises most of the dataset. Improving performance over the minority class is a common challenge in text classification. The most common and effective strategy is perhaps to annotate more data manually so that the system can better generalize the features of the minority class instances. Our postclassification analyses show that the performance of the best classifier does not fully plateau with the data that is currently available for training, meaning that further annotation of data is likely to improve performance albeit only slightly. Another strategy that can be employed to improve classification performance is ensembling. In recent research, we have obtained performance improvements in social media text classification by applying fusion-based classification strategies [25]. We will attempt to apply similar strategies for this task, although, due to the high computational cost associated with running multiple classifiers in an ensemble, improving classification via further annotation is a more sustainable long-term approach.

Another major limitation is associated with the platform—social media subscribers tend to be younger and often more tech-savvy compared to the general population. Thus, Twitter is not fully reflective of the U.S. or global population. It is likely that our cohort is skewed toward younger people and has an underrepresentation of older people. Recent surveys have shown that there is substantial variance in demographic characteristics across social networks [22], and thus, combining data or building a cohort from multiple social networks could lead to the creation of a better representative cohort. Collecting, processing, and aggregating data across multiple social networks is, however, a substantially more challenging task compared to the task described in this paper, and we leave such efforts for future work. Furthermore, only information publicly shared by our cohort is available for analysis. Self-reported information may be biased, and information never shared by a subscriber will thus be missing. The former limitations are not unique to Twitter or social media—no data source is bias-free. Social media, however, is perhaps the platform with the best reach of any. It is also possible that certain population groups will share more information over social media compared to others (e.g., women have been found to share more chronic pain-related information compared to men), and we did not adjust for that. While it is not possible for us to influence the demographic distribution of Twitter, in the future we will use automatic cohort characterization strategies for estimating the distributions of race, gender identity, and age-group of the cohort [46]. A careful study of the biases present in our social media cohort data will also be important future work [47]. Importantly, future studies relying on or utilizing our outputs/findings should be mindful of the many and evolving biases in social media data and the potential harms of propagating inequity.

### Conclusion

Our work is the first that builds an elaborate NLP and machine learning pipeline to curate knowledge about chronic pain from patient-generated social media data. The collection of cohort members via classification and their posts over time is fully automated, and so the cohort and the data will continue to grow over time. This will lead to the creation of an unprecedented resource for conducting long-term studies on the topic. With the detrimental public health impact of opioid pain relievers now well known, our social media mining infrastructure will be of particularly high utility for generating hypotheses about opioid alternatives from the Twitter cohort. Public release of our annotated data will enable the reproduction of this work and the deployment of this pipeline by other researchers. All pretrained models used in this study are publicly available.

## Data Availability

The tweet IDs and their labels are available (Supplementary material S2). Researchers may download the texts of the tweets associated with the IDs as long as they are publicly available via the Twitter API.

## Supplementary Materials

Supplementary 1 Supplementary material S1. Annotation guideline for manually identifying self-disclosures of chronic pain.

Supplementary 2 Supplementary material S2. IDs and labels for the training, development validation, and test sets.

## References

[1] Majeed MH, Ali AA, Sudak DM. Mindfulness-based interventions for chronic pain: Evidence and applications. Asian J Psychiatr. 2018;32:79–83. 29220782 10.1016/j.ajp.2017.11.025

[2] Bauer BA, Tilburt JC, Sood A, Li G-X, Wang S-H. Complementary and alternative medicine therapies for chronic pain. 2016;22(6):403–411. 10.1007/s11655-016-2258-y 27339090

[3] Stoicea N, Costa A, Periel L, Uribe A, Weaver T, Bergese SD. Current perspectives on the opioid crisis in the US healthcare system: A comprehensive literature review. Medicine (Baltimore). 2019;98(20):Article e15425. 31096439 10.1097/MD.0000000000015425 PMC6531094

[4] Nahin RL. Estimates of pain prevalence and severity in adults: United States, 2012. J Pain. 2015;16(8):769–780. 26028573 10.1016/j.jpain.2015.05.002 PMC4562413

[5] Stanos S, Brodsky M, Argoff C, Clauw DJ, D’Arcy Y, Donevan S, Gebke KB, Jensen MP, Clark EL, McCarberg B, et al. Rethinking chronic pain in a primary care setting. Postgrad Med. 2016;128(5):502–515. 27166559 10.1080/00325481.2016.1188319

[6] Urits I, Schwartz EH, Orhurhu V, Maganty NV, Reilly BT, Patel PM, Wie C, Kaye AD, Mancuso KF, Kaye AJ, et al. A compcehensive review of alternative therapies for the management of chronic pain patients: Acupuncture, tai chi, osteopathic manipulative medicine, and chiropractic care. Adv Ther. 2021;38(1):76–89. 33184777 10.1007/s12325-020-01554-0 PMC7854390

[7] Yang Y, Maher DP, Cohen SP. Emerging concepts on the use of ketamine for chronic pain. 2020;13(2):135–146. 10.1080/17512433.2020.1717947 31990596

[8] Brandow AM, Carroll CP, Creary S, Edwards-Elliott R, Glassberg J, Hurley RW, Kutlar A, Seisa M, Stinson J, Strouse JJ, et al. American Society of Hematology 2020 guidelines for sickle cell disease: Management of acute and chronic pain. Blood Adv. 2020;4(12):2656–2701. 10.1182/bloodadvances.2020001851 PMC7322963 32559294

[9] Berger AA, Keefe J, Winnick A, Gilbert E, Eskander JP, Yazdi C, Kaye AD, Viswanath O, Urits I. Cannabis and cannabidiol (CBD) for the treatment of fibromyalgia. Best Pract Res Clin Anaesthesiol, 631. 2020;34(3):617–631. 33004171 10.1016/j.bpa.2020.08.010

[10] Kinney M, Seider J, Beaty AF, Coughlin K, Dyal M, Clewley D.The impact of therapeutic alliance in physical therapy for chronic musculoskeletal pain: A systematic review of the literature. Physiother Theory Pract. 2018;36(8):886–898. 30265840 10.1080/09593985.2018.1516015

[11] Sarker A, O’Connor K, Ginn R, Scotch M, Smith K, Malone D, Gonzalez G. Social media mining for toxicovigilance: Automatic monitoring of prescription medication abuse from twitter. Drug Saf. 2016;39(3):231–240. 26748505 10.1007/s40264-015-0379-4 PMC4749656

[12] Chary M, Genes N, Giraud-Carrier C, Hanson C, Nelson LS, Manini AF. Epidemiology from tweets: Estimating misuse of prescription opioids in the usa from social media. J Med Toxicol. 2017;13(4):278–286. 28831738 10.1007/s13181-017-0625-5 PMC5711756

[13] Sarker A, Gonzalez-Hernandez G, Ruan Y, Perrone J. Machine learning and natural language processing for geolocation-centric monitoring and characterization of opioid-related social media chatter. JAMA Netw Open. 2019;2(11):Article e1914672. 31693125 10.1001/jamanetworkopen.2019.14672 PMC6865282

[14] Spadaro A, Sarker A, Hogg-Bremer W, Love JS, O’Donnel N, Nelson LS, Perrone J. Reddit discussions about buprenorphine associated precipitated withdrawal in the era of fentanyl. Clin Toxicol (Phila). 2021;60(6):694–701. 10.1080/15563650.2022.2032730 PMC10457147 35119337

[15] Sarker A, Ginn R, Nikfarjam A, O’Connor K, Smith K, Jayaraman S, Upadhaya T, Gonzalez G. Utilizing social media data for pharmacovigilance: A review. J Biomed Inform. 2017;54:202–212. 10.1016/j.jbi.2015.02.004 PMC4408239 25720841

[16] Sloane R, Osanlou O, Lewis D, Bollegala D, Maskell S, Pirmohamed M. Social media and pharmacovigilance: A review of the opportunities and challenges. Br J Clin Pharmacol. 2015;80(4):910–920. 26147850 10.1111/bcp.12717 PMC4594734

[17] Arnoux-Guenegou A, Girardeau Y, Chen X, Deldossi M, Aboukhamis R, Faviez C, Dahamna B, Karapetiantz P, Guillemin-Lanne S, Lillo-Le Louet A, et al. The adverse drug reactions from patient reports in social media project: Protocol for an evaluation against a gold standard. JMIR Pre Protoc. 2019;8(5):Article e11448. 10.2196/11448 PMC6528435 31066711

[18] Conway M, Hu M, Chapman WW. Recent advances in using natural language processing to address public health research questions using social media and consumergenerated data. Yearb Med Inform. 2019;28(1):208–217. 31419834 10.1055/s-0039-1677918 PMC6697505

[19] De Choudhury M, Kiciman E, Dredze M, Coppersmith G, Kumar M. Discovering shifts to suicidal ideation from mental health content in social media. Proc SIGCHI Conf Hum Factor Comput Syst. 2016;2016:2098–2110. 29082385 10.1145/2858036.2858207 PMC5659860

[20] Giustini DM, Ali SM, Fraser M, Boulos MNK. Effective uses of social media in public health and medicine: a systematic review of systematic reviews. Online J Public Health Inform. 2018;10(2):Article e215. 30349633 10.5210/ojphi.v10i2.8270 PMC6194097

[21] Pew Research Center. Demographics of Social Media Users and Adoption in the United States. Pew Research Center. 2021. Washington, USA

[22] Pew Research Center. Who uses YouTube, WhatsApp and Reddit. Pew Research Center. 2019. Washington, USA

[23] Al-Garadi MA, Yang Y-C, Lakamana S, Lin J, Li S, Xie A, Hogg-Bremer W, Torres M, Banerjee I, Sarker A. Automatic Breast Cancer Cohort Detection from Social Media for Studying Factors Affecting Patient-Centered Outcomes. AIME 2020; Springer, Cham; 26 Sep 2020.

[24] Sarker A, Chandrashekar P, Magge A, Cai H, Klein A, Gonzalez G. Discovering cohorts of pregnant women from social media for safety surveillance and analysis. J MED Internet Res. 2017;19(10):Article e361. 29084707 10.2196/jmir.8164 PMC5684515

[25] Al-Garadi MA, Yang Y-C, Cai H, Ruan Y, O’Connor K, Graciela G-H, Perrone J, Sarker A. Text classification models for the automatic detection of nonmedical prescription medication use from social media. BMC Med Inform Decis Mak. 2021;21(21):Article 27. 10.1186/s12911-021-01394-0 PMC7835447 33499852

[26] De Nardi L, Trombetta A, Ghirardo S, Genovese MRL, Barbi E, Taucar V. Adolescents with chronic disease and social media: a cross-sectional study. Arch Dis Child. 2020;105(8):744–748. 31941715 10.1136/archdischild-2019-317996

[27] Merolli M, Gray K, Martin-Sanchez F. Health outcomes and related effects of using social media in chronic disease management: A literature review and analysis of affordances.J Biomed Inform. 2013;46(6):957–969. 23702104 10.1016/j.jbi.2013.04.010

[28] Ressler PK, Bradshaw YS, Gualtieri L, Chui KKH. Communicating the experience of chronic pain and illness through blogging. J Med Internet Res. 2012;14(5):Article e143. 23092747 10.2196/jmir.2002 PMC3510726

[29] Tsai S, Crawford E, Strong J. Seeking virtual social support through blogging: A content analysis of published blog posts written by people with chronic pain. Digit Health. 2018;4:Article 2055207618772669. 29942635 10.1177/2055207618772669 PMC6016559

[30] Gonzalez-Polledo E. Chronic media worlds: Social media and the problem of pain communication on tumblr. Soc Media+Society. 2016;2(1).

[31] Sendra A, Farré J. Communicating the experience of chronic pain through social media: patients’ narrative practices on Instagram. J Commun Healthc. 2020;13(1):46–54.

[32] Mullins CF, Ffrench-O'Carroll R, Lane J, O'Connor T. Sharing the pain: An observational analysis of Twitter and pain in Ireland. Reg Anesth Pain Med. 2020;45(8):597–602. 32503862 10.1136/rapm-2020-101547

[33] Twitter. Twitter API for Academic Research, Products. Twitter Developer Platform. 2022. [accessed 1 September 2022] https://developer.twitter.com/en/products/twitter-api/academic-research

[34] Liu Y, Ott M, Goyal N, Du J, Joshi M, Chen, Levy O, Lewis M, Zettlemoyer L, Stoyanov V. RoBERTa: A robustly optimized BERT pretraining approach. ArXiv. 2019. 10.48550/arXiv.1907.11692

[35] Beltagy I, Lo K, Cohan A. SCIBERT: A pretrained language model for scientific text. ArXiv. 2019. 10.48550/arXiv.1903.10676

[36] Alsentzer E, Murphy JR, Boag W, Weng W-H, Jin D, Naumann T, McDermott MBA. Publicly Available Clinical BERT Embeddings. ArXiv. 2019. 10.48550/arXiv.1904.03323

[37] Nguyen DQ, Vu T, Tuan Nguyen A. BERTweet: A pre-trained language model for English Tweets. ArXiv. 2020. 10.48550/arXiv.2005.10200

[38] Lee J, Yoon W, Kim S, Kim D, Kim S, So CH, Kang J. BioBERT: A pre-trained biomedical language representation model for biomedical text mining. Bionformatics. 2019;36(4):1234–1240. 10.1093/bioinformatics/btz682 PMC7703786 31501885

[39] Hao B, Zhu H, Paschalidis I. Enhancing clinical BERT embedding using a biomedical knowledge base. Paper presented at: 28th International Conference on Computational Linguistics, International Committee on Computational Linguistics; 2020 Dec 1; Barcelona, Spain.

[40] Efron B. *Bootstrap methods: Another look at the Jackknife*. Springer Series in Statistics. New York (NY): Springer; 1979

[41] Sarker A, Lakamana S, Hogg-Bremer W, Xie A, Al-Garadi MA, Yang Y-C. Self-reported COVID-19 symptoms on Twitter: An analysis and a research resource. J Am Med Inform Assoc. 2020;27(8):1310–1315. 32620975 10.1093/jamia/ocaa116 PMC7337747

[42] Hutto C, Gilbert E. VADER: A parsimonious rule-based model for sentiment analysis of social media text. Proceedings of the International AAAI Conference on Web and Social Media. 2014;8(1), 216–225.

[43] Cohen J. A coefficient of agreement for nominal scales. Edu Pschol Measur. 1960;20(1):37–46.

[44] Viera AJ, Garrett JM. Understanding interobserver agreement: The kappa statistic. Fam Med. 2005;37(5):360–363. 15883903

[45] Sarker A, Gonzalez G. Portable automatic text classification for adverse drug reaction detection via multi-corpus training.J Biomed Inform. 2015;53:196–207. 25451103 10.1016/j.jbi.2014.11.002 PMC4355323

[46] Yang Y-C, Al-Garadi MA, Love JS, Cooper HLF, Perrone J, Sarker A. Can accurate demographic information about people who use prescription medications nonmedically be derived from twitter? Proc Natl Acad Sci. 2023;120(8):Article e2207391120. 36787355 10.1073/pnas.2207391120 PMC9974473

[47] Zhao Y, He X, Feng Z, Bost S, Prosperi M, Wu Y, Guo Y, Bian J. Biases in using social media data for public health surveillance: A scoping review. Int J Med Inform. 2022;164:Article 104804. 35644051 10.1016/j.ijmedinf.2022.104804 PMC12905649

