# Supplementary material for: #ChronicPain: Automated Building of a Chronic Pain Cohort from Twitter Using Machine Learning
Source: Health Data Sci. Author manuscript; Available in PMC 2024 Feb 8. (PMC10852024; doi:10.34133/hds.0078)
Supplement: supplment 1 — Supplementary material S1. Annotation guideline for manually identifying self-disclosures of chronic pain. [file NIHMS1926164-supplement-supplment_1.docx]

# **Chronic pain classification from social media: Annotation guidelines**

Version: 2.0

Last updated: September 03, 2022

**Purpose**

The goal of the annotation task is to label tweets as one of two categories: (i) *self-report of chronic pain* and (ii) *general chronic pain chatter (not first-person report).* The following guidelines will help annotators, with examples, to determine which tweets should be labeled as self-reports of chronic pain or first-hand reports of chronic pain.

**Introduction**

Our annotation will classify the tweets into two categories:

1. *Self-report of chronic pain (Y)*: These tweets contain possible indications that the users are referring to their own personal experience of suffering from chronic pain. This may include references to current chronic pain or past experiences of chronic pain. Chronic pain suffered by a family member or a third person related to the user is not considered to be a self-report. There must be some indication that the experience being represented is the person’s own experience, although explicit mention is not required. Expressions that imply that the chronic pain experience is first-hand experience should be in this category.
2. *General chronic pain chatter (N).* In these tweets the mention of the hashtag #chronicpain or the phrase *chronic pain* is not associated with any first-hand experience of chronic pain. These tweets may be sharing chronic pain related information or news about therapies, medications, jokes, movie or book titles, lines from movies or songs, etc.

We now provide some examples of annotations.

**1. Chronic pain self-reports (Y)**

The following are some examples of self-reports of chronic pain.

1. ADHD mixed with chronic pain/chronic illness is a special kind of hell I must do everything, ooo shiny, mixed w/ random fatigue and pain is a recipe for disaster lol
2. #ADHD #adhdtwitter #NEISvoid #chronicpain #chronicillness #Fibromyalgia #adhdfolk #DisabilityTwitter
3. Good luck with that!! I have 3 herniated discs, sitting on nerves, spinal hypertrophy, chronic pain...diagnosed 7 months ago and my appt to see an NHS specialist THIS Oct has been cancelled Now on the waiting list No end in sight!!! Hope u have better luck #ChronicPain
4. 26 years old, 15 years of chronic pain and fatigue... finally diagnosed with Endometriosis #awareness #endometriosis #ChronicPain
5. My chronic pain is gotten worse since my covid infection. I wake up with pain in my neck muscles every day now.
6. Chronic pain will have you thinking the craziest thing. My hips are killing me so I thought it might be nice to not have legs…. But then I realise my back and arms also hurts so then I thought what about being a floating head… then I remembered migraines. #chronicpain

**2. General chronic pain chatter (N)**

1. "Plant-derived compound may help treat chronic pain. #chronicpain #CRP [LINK]"
2. "Chronic pain and low oxalate diet? Some people find a huge amount of relief from trying a low oxalate diet when they have chronic pain. Have you tried it? Let me know #chronicpain #spoonie #chronicillness #cfs #fibro"
3. ADHD mixed with chronic pain/chronic illness is a special kind of hell 😝 I must do everything, ooo shiny, mixed w/ random fatigue and pain is a recipe for disaster lol #ADHD #adhdtwitter #NEISvoid #chronicpain #chronicillness #Fibromyalgia #adhdfolk #DisabilityTwitter
4. "To know that no one will help you. Bc ""they don't want to step only anyone's toes"" so you just get passed around from doctor to doctor. This is why chronic pain patients are pissed about this! #chronicillness #chronicpain #Doctor #pain #MedTwitter #DisabilityTwitter #thread"
5. "Chronic pain, PTSD top the list of reasons people buy medical marijuana in Illinois. Number of medicinal cardholders grows by 30%.
6. #MedicalMarijuana #MedicalCannabis #Marijuana #Cannabis #Health #MentalHealth #ChronicPain #PTSD #Illinois [LINK]
